# The Effects of a 10-Week Neuromuscular Training on Postural Control in Elite Youth Competitive Ballroom Dancers: A Randomized Controlled Trial

**DOI:** 10.3389/fphys.2021.636209

**Published:** 2021-03-25

**Authors:** Meiqi Zhang, Hongtao Ma, Zhan Liu, Daniel M. Smith, Xiao Wang

**Affiliations:** ^1^Arts School, Beijing Sport University, Beijing, China; ^2^Department of Physical Education and Health Education, Springfield College, Springfield, MA, United States

**Keywords:** neuromuscular training, ballroom dance, dancer, postural control, athletic performance

## Abstract

The purpose of this randomized controlled trial was to evaluate the efficacy of a 10-week neuromuscular training (NMT) program on the postural control of elite youth competitive ballroom dancers. Forty-two dancers (21 couples) were randomly assigned to either the NMT group (*n* = 22) or the control group (CG; *n* = 20). Participants in NMT underwent a three-sessions-per-week NMT program for 10 weeks. Testing at baseline and after the 10 weeks intervention included the Y-balance test (YBT) and Modified-Balance Error Scoring System (M-BESS). Results of YBT indicated that NMT participants demonstrated increased reach in the posterolateral and posteromedial directions for the right and left lower limb, whereas no significant change was found in the anterior direction for both limbs. Results of Modified-Balance Error Scoring System (M-BESS) showed that NMT participants displayed significantly decreased errors of the double-leg floor (*p* = 0.026), single-leg foam (*p* = 0.010), double-leg foam (*p* = 0.003), tandem floor (*p* = 0.031), and tandem foam (*p* = 0.038), while no significant change was found in single-leg floor performance (*p* = 0.476). CG participants did not exhibit any significant change during the 10-week period. In summary, the study affirmed that the 10-week NMT program enhanced the postural control performance of youth ballroom dancers and showed effects on ballroom dance-specific performance and lower-limb injury prevention. The results suggest that NMT may be a valuable addition to ballroom dance training regimens.

## Introduction

Competitive ballroom dance, also known as international standard dance or smooth dance, consists of five dances, which are Waltz, Tango, Viennese Waltz, Foxtrot, and Quickstep (WDSF, 2018). The five dances are overall characterized by couples move rhythmically to display the elegance and fluidity of movement and express the feature of music; while the difference in technical requirements of the five dances is obvious, in particular to postural control performance. Postural control could be defined as the act of maintaining, achieving or restoring a state of balance during any posture or activity (Pollock et al., 2000). To ballroom dance, Viennese-Waltz mainly emphasizes maintaining stably posture during continuous pivoting (achievement); Quickstep challenges dancers to perform accurate control to fast walking, jumping, and chasseing steps (maintenance). As Prosen et al. (2013) suggested, the ability to maintain a high-speed movement in the more difficult turns, particularly the reverse turns, is a critical factor to differentiate higher- and lower-ranked elite ballroom dancers. In addition, the poorly predictable environment of competitions (i.e., six to eight couples dance on a rectangular floor simultaneously but moving tracks are different) and interaction between partners (restoration) also raised higher demands for the postural control of dancers. Superior and comprehensive postural control is thus an important component of the skills required for elite ballroom dancers. However, to date, no study was found in the literature that focused on how to improve the ability of ballroom dancers specifically.

As a non-traditional form of training, neuromuscular training (NMT) is receiving increasing research attention in the sports field, mainly because NMT is purported to be effective for the rehabilitation of sports injuries (Zech et al., 2009a) and prevention of lower limb injuries (Hübscher et al., 2010), through its unique capacity of provoking physiological sensory feedback alterations and therefore enhance joint functionalities (Hewett et al., 1999; Coughlan and Caulfield, 2007; Zech et al., 2009b; Myer et al., 2013). The effects of NMT on improving sport performance had also been examined in various physical fitness components, including jumping (Chappell and Limpisvasti, 2008), agility (Zouhal et al., 2019), abdominal endurance (Barber-Westin et al., 2015), and postural control performance (Paterno et al., 2004; Filipa et al., 2010; Asadi et al., 2015; Benis et al., 2016; Zemková and Hamar, 2018).

Considering the significant role of postural control to ballroom dancers and the efficacy of NMT, we conducted a randomized controlled trial of the effects of NMT on postural control performance in competitive ballroom dancers. The primary purpose of the study was to evaluate the efficacy of a 10-week NMT program on the postural control performance in a sample of youth elite competitive ballroom dancers, as assessed with Y-balance test (YBT) and M-BESS. Two secondary objectives of the study were to (1) detect the sport-specific effects of the NMT program, and (2) determine the preventive effects of lower-limb injuries. The main hypotheses of the study were that NMT will enhance balance performance and reduce the risk of lower limb injuries.

## Materials and Methods

### Experimental Approach to the Problem

A randomized controlled trial was conducted with concealed allocation and blinded outcome assessment. Eligible 42 elite youth ballroom dancers (21 couples) were randomly assigned to either the NMT group or the control group (CG). Random assignment was implemented by the random number table method, in which male athletes drew sealed numbers produced on a laptop to determine if they would be assigned to either NMT or CG with their dance partners. Outcome measures were conducted within 1 week before and after the 10-week training intervention. All the participants gave written informed consent to participate, and the study protocol was approved by the Institutional Ethics Review Committee of the Beijing Sport University (Beijing, China) and conducted in accordance with current Chinese and international laws and regulations governing the use of the Declaration of Helsinki II.

### Subjects

Elite collegiate ballroom dancers recruited from the arts school of Beijing Sport University through training venues poster, advertisement and coaches’ notice between September 15 and September 30, 2017. Study participants were eligible to participate in this study if they met the following criteria: (1) they were able to participate in the study with their constant dancing partner; (2) they provided informed consent; (3) they had no chronic ankle instability, anterior cruciate ligament, or any lower extremity injury (overuse or acute) over the past six months. All the participants were required to keep the regular curriculum and ballroom dance training (2 h/time, 5 times/week) but not engage in or do any other forms of physical training. Data on medical history, age, height, length of lower limb, body mass, training experience, injury history, and performance level were collected at baseline (Table 1).

**TABLE 1:** Demographic characteristics of the participants at the baseline.

	**NMT (*n* = 22)**		**CG (*n* = 20)**	
	**Male**	**Female**	**Male**	**Female**
	**(*n* = 11)**	**(*n* = 11)**	**(*n* = 10)**	**(*n* = 10)**
Age (years)	19.81 ± 1.72	19.02 ± 1.97	20.8 ± 1.24	20.9 ± 1.58
Height (cm)	177.57 ± 6.43	164.04 ± 6.79	176.69 ± 7.54	165.77 ± 5.02
Lower-limb length (cm)	103.1 ± 3.23	91.79 ± 4.02	102.44 ± 3.42	90.78 ± 3.77
Mass (kg)	69.32 ± 9.36	51.71 ± 8.21	72.47 ± 9.81	52.86 ± 7.90
Training age (years)	13.92 ± 2.89	13.21 ± 3.75	14.71 ± 3.62	14.13 ± 4.13

*NMT = neuromuscular training group; CG = control group.*

### Procedures

**FIGURE 1:**
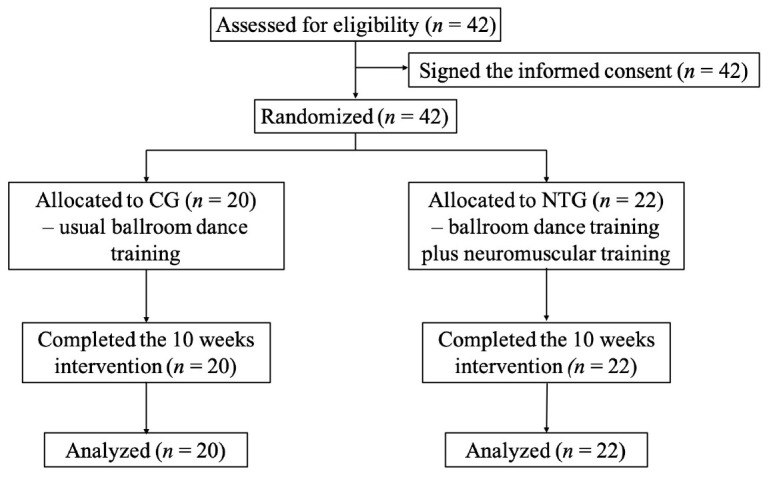
Study flow diagram (NMT = neuromuscular training group; CG = control group).

The whole process is shown in the Figure 1. During the intervention, participants in the CG were required to not attend any other physical exercise. The participants in the NMT group performed 60 min per session, and three sessions per week of the NMT that were led by experienced instructors. Before the formal intervention, participants in the NMT group were provided with three sessions weekly for two weeks to study the movements and drills from specialized instructors. Additionally, participants in the NMT group were required to submit journal entries weekly for instructors knowing their feelings with the intervention (e.g., the difficulty and intensity) and making adjustments on time.

The NMT program consisted of three periods (week 1–3, week 4–7, and week 8–10). Motor proficiency and details were emphasized more at the initial phase, and then the difficulty of practice was gradually increased. Each session of the NMT consisted of a 10-min ballroom dance-specific warm up, a 45-min NMT (Table 2) and followed by a 5-min stretching and closure. The NMT protocol was developed on the basis of communications with the experts in fitness training and dance coaching, and closely connected to the technical requirements of ballroom dancing and the potential advantages of NMT.

**TABLE 2:** Neuromuscular training program (repetitions/duration × sets).

**Periods**	**Week 1–3**	**Week 4–7**	**Week 8–10**
**Surface**			
**Practice by one**			
Balance disc	Lunge 30 s/leg × 3	Backward Lunge 10/leg × 3	Backward lunge and back to balance 10 reps/leg × 3
BOSU ball (reversed)	Narrow standing with posture keeping 30 s × 3	Tandem standing with posture keeping 30 s × 3	Single leg standing with posture keeping 30 s/leg × 3
	Static squat 30 s × 3	Bodyweight squat 15 reps × 3	Squat with holding a med-ball 15 reps × 3
**Practice by partners**			
Balance disc	Med-ball catch and throw with double leg standing 2 min × 2	Med-ball catch and throw with single leg standing 2 min × 2	Med-ball catch and throw with tandem standing 2 min × 2
Balance foam pat	Weight shifts with holding hands* 1 min × 3	Weight shifts with posture holding* 1 min × 3	Swaying with posture holding* 1 min × 3
	Body rotation with holding hands* 1 min × 3	Rotation with posture holding* 1 min × 3	Rotation with posture holding 2s* 1 min × 3
	Front-side-reverse leg lift up and down with holding hands* 30 s/leg × 3	Front-side-reverse leg lift up and down with posture holding* 30 sec/leg × 3	Front-side-reverse leg lift up and down with posture holding* 1 min/leg × 3

All the ballroom dancing-specialized practices (marked as *) were completed with ballroom dancing music.

### Measurements

Before testing, the participants were provided verbal instructions and watched a video demonstration, followed by 10 min of warm-up and 5 min of basic footwork exercise (both were ballroom dance specialized). However, stretching movements were not permitted. All the trials required participants to be barefoot and wearing lightweight clothing.

#### Modified-Balance Error Scoring System

During the M-BESS, participants were required to stand in front of an anthropometric grid and hold upper the body as ballroom posture rather than the original version of the M-BESS, which maintains hands on the hips. Six experimental trials were performed with the eyes closed in the following stances: double leg on the floor, single leg on the floor, tandem on the floor, double leg on a foam pad, single leg on a foam pad, and tandem on a foam pad. Each trial was performed for 20 s, during which time the rater recorded errors. Errors were determined as opening eyes, moving arm(s), lifting heel(s), step stumbles or fall, abduction or flexion of the hip beyond 30°, and any other remaining out of the proper testing position. If there were two errors committed simultaneously, it was counted as a single error (Howell et al., 2017). Therefore, a lower M-BESS result would indicate that the subjects demonstrated better static balance ability.

#### Y-Balance Test

The YBT has the participant stand on the stance platform of the Y-shaped test equipment with one leg while using the other leg to sequentially push the calipers in three different directions (i.e., anterior, posteromedial, and posterolateral) as far as possible. During the trials, participants were required to complete three times in each direction with each leg. Touching the floor and balance using a support pole were not allowed. The maximum scores for each direction were recorded. Scores of composite reach distance were calculated by sum of the three reach directions.

### Data Analysis

Data normality and homogeneity of variance were checked before conducting statistical analysis. Categorized variables are described by frequencies, whereas continuous variables are presented as mean ± standard deviation (SD). The intraclass correlation coefficient (ICC) with a two-way random effects model and 95% confidence interval (CI) was computed to evaluate the reliability of M-BESS and YBT. The ICC for the baseline and post-testing M-BESS was 0.763 (95% CI [0.599–0.865]), *p* < 0.001. The ICC for the baseline and post-testing YBT was 0.754 (95% CI [0.543–0.868]), *p* < 0.001. To determine the efficacy of NMT on the YBT performance, 2 × 2 (Group × Time) factorial analysis of variance (ANOVA) was conducted. The two-tailed Independent sample *t*-test was used to demonstrate the changes in M-BESS tests between the baseline and the posttest. The Mann–Whitney *U*-test was used for variables violating assumptions. Significance was assumed at *p* value ≤ 0.05 (two-tailed). R (R-Core-Team., 2020) in RStudio (RStudio Team, 2020), mainly the R packages “bruceR” (Bao, 2020) was used to analyze the collected data. GraphPad Prism 8 (San Diego, CA, United States) was performed for Statistical analysis.

## Results

### Y-Balance Test

The two-way factorial ANOVA was conducted to examine the influence of two independent variables (i.e., time and intervention) on the performance of YBT. Time included two levels (i.e., before and after intervention), and intervention also consisted of two levels (i.e., NMT and CG). Levene’s test of sphericity was non-significant (*p* = 0.42 for the right direction and *p* = 0.07 for the left direction); therefore, the homogeneity of variance assumption was satisfied.

**FIGURE 2:**
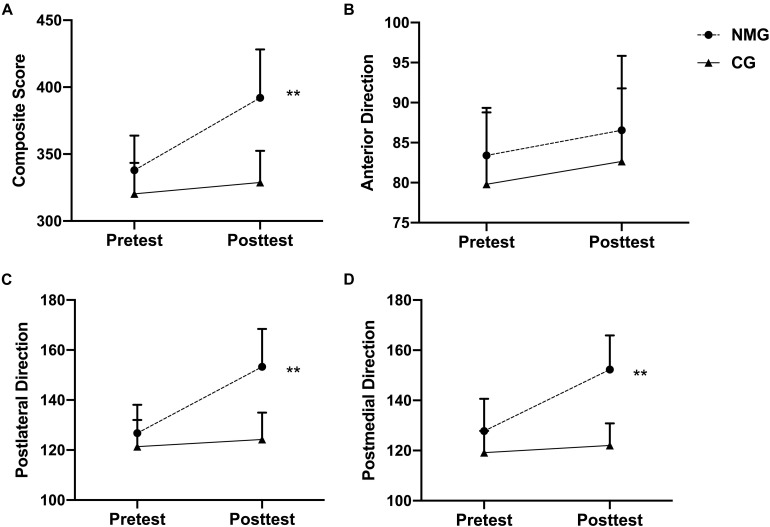
Changes in the Composite **(A)**, the anterior- **(B)**, posterolateral- **(C)**, and posteromedial- **(D)** direction Y-balance test score on right limb in the neuromuscular training group (NMT) and the control group (CG) before and after the program (**inter group difference with *p* ≤ 0.01).

The results of the two-way factorial ANOVA showed a significant interaction between group and time in the composite score of right-limb YBT (Figure 2A), *F*(1,80) = 14.024, *p* < 0.001, η^2^ = 0.149, indicating that the NMT participants had a better average performance on the composite score of YBT on right limb after the intervention compared with the CG. Simple effects tests were performed as *post hoc* analysis for the significant interaction. No significant mean difference (*ps* > 0.05) was found in the baseline condition. However, the NMT scored significantly higher at the posttest (392 ± 36) than the CG (329 ± 23), *t*(80) = 7.347, *p* < 0.001, *d* = 3.015. For the anterior direction of YBT on right limb, there was no significant interaction observed (Figure 2B), *F*(1,80) = 0.006, *p* = 0.938, η^2^ = 0.001. The main effect for time yielded an F ratio of *F*(1,80) = 2.648, *p* = 0.108, η^2^ = 0.032, indicating that the effect for pretest (*M* = 83, *SD* = 6) and posttest (*M* = 86, *SD* = 9) was not significant; while the main effect for group yielded an F ratio of *F*(1,80) = 4.162, *p* = 0.054, η^2^ = 0.049, indicating no significant difference between the NMT and the CG (i.e., 79 ± 9 and 82 ± 9). A significant interaction was observed in the posterolateral direction of right limb YBT (Figure 2C), *F*(1,80) = 19.680, *p* < 0.001, η^2^ = 0.197, indicating that the NMT participants had better average performance after the intervention in comparison to the baseline also the CG. The results of a simple effect test demonstrated that no significant mean difference (*ps* > 0.05) was found in the baseline condition. However, the NMT scored significantly higher at the posttest (153 ± 15) than the CG (124 ± 10), *t*(80) = 7.772, *p* < 0.001, *d* = 3.353. For the posteromedial direction of right limb YBT, a significant interaction was observed (Figure 2D), *F*(1,80) = 19.124, *p* < 0.001, η^2^ = 0.193, which means that the NMT participants got higher average scores in the posttest in comparison to the baseline also the CG. The results of a simple effect analysis showed that no significant mean difference (*ps* > 0.05) was found in the baseline condition, but the NMT scored significantly higher at the posttest (152 ± 13) than the CG (122 ± 8), *t*(80) = 8.649, *p* < 0.001, *d* = 4.023.

**FIGURE 3:**
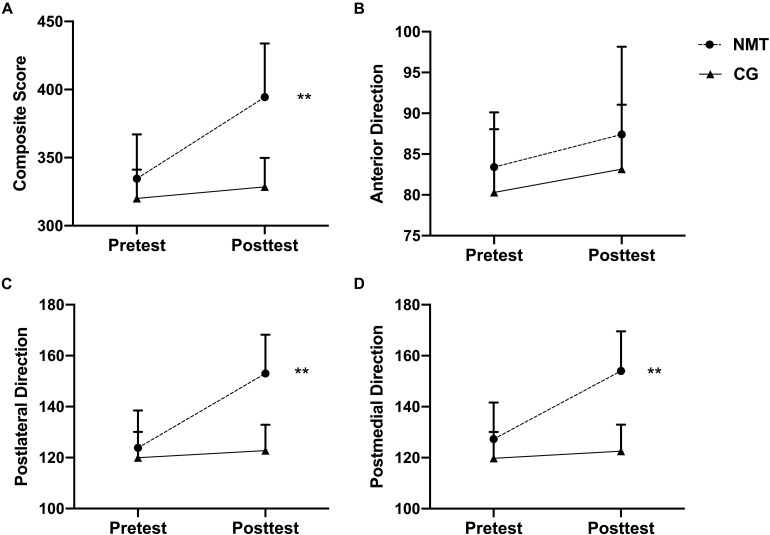
Changes in the Composite **(A)**, the anterior- **(B)**, posterolateral- **(C)**, and posteromedial- **(D)** direction Y-balance test score on left limb in the neuromuscular training group (NMT) and the control group (CG) before and after the program (**inter group difference with *p* ≤ 0.01).

Using 2 × 2 (Time × Group) ANOVA, a significant interaction between group and time in the composite score of left-limb YBT was observed (Figure 3A), *F*(1,80) = 15.357, *p* < 0.001, η^2^ = 0.161, indicating that the NMT participants had a better average performance on the composite score of the left-limb YBT in comparison with the baseline and the CG. A follow-up simple effect test showed that no significant mean difference (*ps* > 0.05) was found in the baseline condition, but the NMT scored significantly higher at the posttest (394 ± 39) than the CG (328 ± 21), *t*(80) = 7.112, *p* < 0.001, *d* = 2.704. For the anterior direction of YBT on the left-limb, there was no significant interaction observed (Figure 3B), *F*(1,80) = 0.097, *p* = 0.756, η^2^ = 0.001. The main effect for time yielded an F ratio of *F*(1,80) = 3.450, *p* = 0.067, η^2^ = 0.041, indicating that the effect for pretest (NMT: 83 ± 6, CG: 80 ± 7) and posttest (NMT: 87 ± 10, CON: 83 ± 8) was not significant; while the main effect for group yielded an F ratio of *F*(1,80) = 3.992, *p* = 0.051, η^2^ = 0.048, indicating no significant difference between NMT and the CG either. A significant interaction was observed in the posterolateral direction of left-limb YBT (Figure 3C), *F*(1,80) = 21.870, *p* < 0.001, η^2^ = 0.215, indicating that the NMT participants had better average performance at the posttest in comparison to the baseline and in comparison, to the CG. The results of a simple effect test demonstrated that no significant mean difference (*ps* > 0.05) was found in the baseline condition. However, the NMT scored significantly higher at the posttest (153 ± 15) than the CG (122 ± 10), *t*(80) = 7.583, *p* < 0.001, *d* = 3.584. For the posteromedial direction of the left-limb YBT, a significant interaction was observed (Figure 3D), *F*(1,80) = 17.736, *p* < 0.001, η^2^ = 0.181, which means that the NMT participants got higher average scores at the posttest in comparison to the baseline also the CG. The results of a simple effect analysis showed that no significant mean difference (*ps* > 0.05) was found in the baseline condition. However, the NMT scored significantly higher at the posttest (127 ± 14) than the CG (119 ± 10), *t*(80) = 7.843, *p* < 0.001, *d* = 1.012. The raw data and the normalized data of YBT are attached in the Supplementary Material.

### Modified-Balance Error Scoring System

**FIGURE 4:**
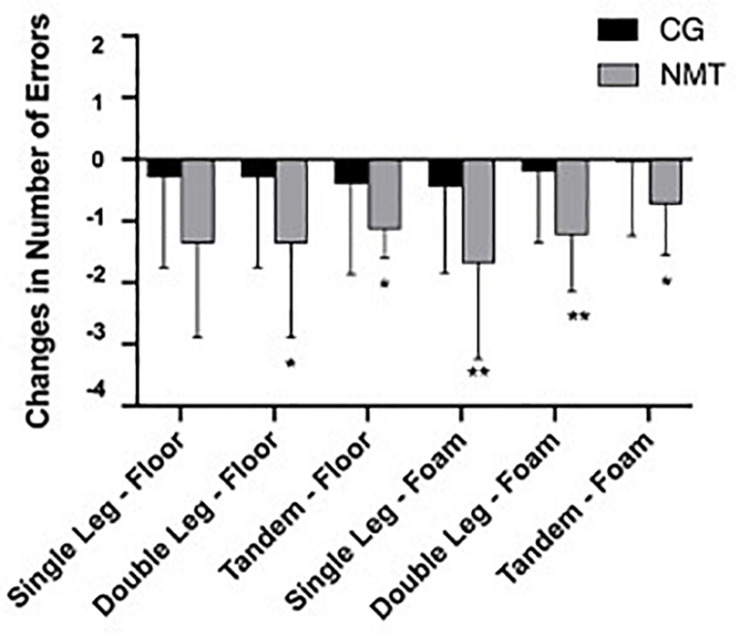
Changes in number of errors of the Balance Error Scoring System test (NMT = neuromuscular training group; CG = control group; *inter group difference with *p* ≤ 0.05; **inter group difference with *p* ≤ 0.01).

By conducting an independent samples *t*-test, we found that participants in the NMT group displayed significant reductions in average errors of the double-leg floor in comparison to the baseline (NMT: −1.364 ± 1.529, CG: −0.300 ± 1.456, *t* = 2.304, *p* = 0.026, *d* = −0.715). Similar findings also were observed in the single-leg foam (NMT: −1.682 ± 1.555, CG: −0.450 ± 1.395, *t* = 2.692, *p* = 0.010, *d* = −0.832), the double-leg foam (NMT: −1.227 ± 0.922, CG: −0.200 ± 1.152, *t* = 3.205, *p* = 0.003, *d* = −0.989); while no significant change was found in the single-leg floor performance (NMT: −1.046 ± 1.496, CG: −0.750 ± 1.118, *t* = 0.719, *p* = 0.476, *d* = 0.223; Figure 4). Because the variances were not equal for the comparisons including the tandem floor and tandem foam, Mann–Whitney *U*-test was used to observe the changes in the two indicators. The results indicated that participants in the NMT group significantly improved performance in the tandem floor (i.e., −1.136 ± 0.468) compared to the CG (i.e., −0.400 ± 1.465), *U* = 161.500, *p* = 0.031, effect size *r* = −0.332. In the tandem foam, similar results were observed (i.e., NMT: −0.727 ± 0.827, CG: −0.050 ± 1.191), *U* = 146.000, *p* = 0.038, effect size *r* = 0.283 (S1).

## Discussion

Competitive ballroom dance challenges the multifaceted postural control ability of dancers – the competitive form, different technique in the five dances, and cohesiveness between partners. Nonetheless, it seemed that no specific training programs have yet been studied for improving postural control performance of competitive ballroom dancers. Previous studies have documented the efficacy of NMT, particularly evident in preventing injuries (Benjaminse et al., 2015) and developing motor performance (Paterno et al., 2004; Filipa et al., 2010). Therefore, we conducted a randomized controlled study that focused on the effects of 10 weeks of an NMT intervention on postural control performance in youth elite ballroom dancers. The main findings were that (1) subjects the NMT performed significant improvement in dynamic postural control performance before and after intervention in comparation to the CG; and (2) with respect to static postural control, as measured by the M-BESS, marked decreases in total M-BESS errors were also found in the NMT group. The findings confirmed the hypothesis of the study that NMT can result in significant gains in overall postural control performance in youth elite ballroom dancers.

The dynamic postural control could be defined as the ability to perform a motor task while maintaining a stable position (Winter et al., 1990), which is an important factor to influence ballroom dance performance. Developed dynamic postural control could not only increase the quality of choreographies but also reduce the risk of lower extremity injuries. After the NMT program, enhanced dynamic postural control performance was detected in the NMT group, which was reflected in the composite score, the posterolateral, and posteromedial direction of YBT on both limbs. The result is consistent with previous studies, which found composite dynamic balance performance gains significantly in athletes who participated in NMT programs (McLeod et al., 2009; Filipa et al., 2010; Benis et al., 2016). Unfortunately, our study failed to provide sufficient evidence to confirm the effects of NMT on anterior YBT, even if an increasing trend was observed after the intervention. This finding was in line with the previous studies (Filipa et al., 2010; Benis et al., 2016), in which no significant differences in anterior reach were detected in basketball and soccer players before and after NMT. In contrast, a recent study by conducting an 8 weeks NMT intervention in skiing athletes had observed significant improvement in anterior YBT performance (Vitale et al., 2018). Some variables, like gender and training protocols, might be identified as the causes of the inconsistent findings. In terms of gender of included participants, the study that observed improvements in anterior reaching (Vitale et al., 2018) included male athletes alone, the aforementioned studies that found no significant improvements in the indicator (Filipa et al., 2010; Benis et al., 2016) only included female athletes. We attempted to analyze the gender difference by using ANOVA, but there was no significant change found. Moreover, differences in training protocols might be another factor leading to the inconsistent results. In our training protocol, there was not enough practice for hip extensor and knee flexor that were examined to be for anterior YBT performance (Lee et al., 2014). Besides, evidence also suggested that the anterior YBT performance is correlated weakly to technical performance (Butler et al., 2016).

In addition to knowing the benefits of the NMT program on postural control, the results may also provide information about the efficacy of reducing lower limb injuries. Due to the requirement of wearing dress/heel shoes in competitions, there are high incidences of lower limb injuries and chronic pains in ballroom dancers, especially on knees (i.e., 40.6% in male, 29.6% in female) and ankles (i.e., 27.0% in male, 24.4% in female) (Miletic et al., 2011). The YBT performance has been widely used as a predictor for evaluating the risk of lower limb injuries (Chimera et al., 2015; Gonell et al., 2015). Therefore, even though the effects of NMT on preventing injuries have not been investigated directly, the increased YBT performance could be regarded as a reference.

Beyond dynamic postural control, static balance would influence athletic performance directly and indirectly. To ballroom dance, in particular Waltz, Viennese Waltz, and Foxtrot choreographies, there are numerous pulsing and stretching movements requiring dancers to maintain posture statically. Static postural control refers to the ability to maintain a base of support with minimal movement (Winter et al., 1990). As a primary result of our study, we observed that the 10 weeks NMT had positive effects on static balance performance in the ballroom dancers, which reflected in the significant reduction of M-BESS errors. The results were expected and in agreements with previous studies showing that NMT improved static balance performance in athletes (McLeod et al., 2009; Zech et al., 2014). Appropriate intensity, frequency, duration and volume of intervention might be a key factor explaining the growth. An early study that had 5 min per session, three sessions per week, and a total of 4 weeks of balance training reported that no significant balance performance differences were found after training within either experimental or CGs (Cox et al., 1993).

Human postural control depends on the integration of the visual system, the vestibular system, and the somatosensory system, each of the systems provides different information to allow the brain and neural system to create necessary responses (Krasnow and Wilmerding, 2015). Adults sustain normal posture and locomotion mainly relying on somatosensory input, especially proprioception, while vision and vestibular function could mediate or take over when proprioception is compromised (Krasnow and Wilmerding, 2015). Postural control performance in dance is constrained by more factors – individual, task, and environmental aspects (Gabbard, 2018) (e.g., the size of the support base). Dancers should have excellent responses in both proprioception and vestibule, as well as visual cues. To ballroom dancers, maintaining a posture by two people is probably the characteristic. This sounds easier, but actually tends to be a greater challenge due to each dancer’s need to give more attention while leading and following with the partner rather than only themselves.

Our observation of the improved postural control performance in dancers could be partially explained by physiological adaptations in response to the NMT. The training-induced adaptive changes are primarily due to neural pruning leading to greater motor efficiency and automation (James et al., 2014). The previous studies also indicated that the observed increases in motor performance in lower limbs were more related to the control of motor neurons to the trunk and muscles in lower limbs, while less related to changes in muscle strength (Kravitz et al., 2003; Filipa et al., 2010; Ozer et al., 2011). Meanwhile, the Hebbian plasticity theory may also help to explain the adaptive changes, in which synaptic efficacy could be strengthened when presynaptic neurons repeatedly and persistently stimulate postsynaptic neurons (Wolters et al., 2003; Takeuchi and Izumi, 2015).

The NMT program used in this study was developed and implemented closely based on the techniques of ballroom dancing, which would offer additional benefits to dance performance. First, the training protocol was explored based on the movement patterns of ballroom dance. Second, an expert dance coach attended each session to supervise and give corrective suggestions to the participants on details and accuracy of techniques. After the 10 weeks NMT program, visual and verbal feedback from the head coaches confirmed the overall improvements in the dance performance of the NMT participants, especially on the execution of basic steps. Lunge drills were involved throughout the NMT program that might help explaining the observable changes in basic steps. Moreover, we separated the 10 weeks training program as three progressive periods. For example, more double-leg practices could be seen in the initial phase of the program, and then we gradually increased the contents of practice with single leg (Myklebust et al., 2003). Such scenarios could better address the details of technique in the NMT program and also reduce the risk of injuries.

However, there are potential limitations in the present study. First, the two groups sustained a different training volume, which may result in bias and confounding impacts to some extent. Second, mixed-gender sport is an important feature of ballroom dance, but gender difference could not be teased apart in the study. Third, because there is no quantitative data (e.g., functional near-infrared spectroscopy), the NMT-induced changes could not be reflected more specifically. Neurophysiological and neuroimaging techniques should be applied to clarify and confirm the mechanisms involved. Fourth, due to the limited duration of the study, how to maintain the improved balance performance is still not very clear, differences between detraining, low-volume training, and continuous training are needed to elucidate.

## Conclusion

The study suggests that ballroom dancers can improve postural control performance by participating in the NMT program. Additional concomitant benefits of NMT through indirect evidence include improving ballroom dance-specific performance and reducing the risks of lower limb injuries, indicating the utility of NMT among competitive ballroom dancers. With this accumulated evidence, the time might be near for NMT to be recommended as an ideal strategy for balance exercise and to be routinely arranged into a regular ballroom dance training regimen.

## Data Availability Statement

The original contributions presented in the study are included in the article/Supplementary Material, further inquiries can be directed to the corresponding author/s.

## Ethics Statement

The studies involving human participants were reviewed and approved by the Beijing Sport University. Written informed consent to participate in this study was provided by the participants’ legal guardian/next of kin.

## Author Contributions

MZ, HM, ZL, DS, and XW conceptualized the study. MZ and XW contributed to collecting the data and drafting the manuscript. ZL and DS contributed to revising and approving the final version of the manuscript. HM contributed to the overall coordination of the trial. All authors contributed to the article and approved the submitted version.

## Conflict of Interest

The authors declare that the research was conducted in the absence of any commercial or financial relationships that could be construed as a potential conflict of interest.
