# Supplementary material for: The Effects of a 10-Week Neuromuscular Training on Postural Control in Elite Youth Competitive Ballroom Dancers: A Randomized Controlled Trial
Source: Front Physiol. 2021 Mar 25;12:636209. doi: 10.3389/fphys.2021.636209 (PMC8027106; doi:10.3389/fphys.2021.636209)
Supplement: Supplementary file 1 [file Data_Sheet_1.docx]

**Raw Data for Y-Balance Test**

|  | **NMT (n=22)** | | | | **CG (n=20)** | | | |
| --- | --- | --- | --- | --- | --- | --- | --- | --- |
|  | **Pretest** | | **Posttest** | | **Pretest** | | **Posttest** | |
|  | **Mean** | **SD** | **Mean** | **SD** | **Mean** | **SD** | **Mean** | **SD** |
| Right anterior | 83.41 | 5.94 | 86.55 | 9.29 | 79.80 | 8.98 | 82.65 | 9.14 |
| Right postlateral | 126.77 | 11.31 | 153.27 | 15.17 | 121.35 | 10.71 | 124.20 | 10.79 |
| Right postmedial | 127.77 | 12.85 | 152.27 | 13.61 | 119.15 | 8.71 | 122.00 | 8.81 |
| Right composite | 337.95 | 25.80 | 392.09 | 36.11 | 320.30 | 23.15 | 328.85 | 23.56 |
| Left anterior | 83.41 | 6.71 | 87.41 | 10.74 | 80.30 | 7.75 | 83.15 | 7.91 |
| Left postlateral | 123.82 | 14.66 | 153.00 | 15.26 | 119.95 | 10.15 | 122.80 | 10.07 |
| Left postmedial | 127.32 | 14.31 | 154.05 | 15.53 | 119.75 | 10.38 | 122.60 | 10.39 |
| Left composite | 334.55 | 32.54 | 394.45 | 39.38 | 320.00 | 21.23 | 328.55 | 21.30 |

**Normalized Data for Y-Balance Test**

|  | **NMT (n=22)** | | | | **CG (n=20)** | | | |
| --- | --- | --- | --- | --- | --- | --- | --- | --- |
|  | **Pretest** | | **Posttest** | | **Pretest** | | **Posttest** | |
|  | **Mean** | **SD** | **Mean** | **SD** | **Mean** | **SD** | **Mean** | **SD** |
| Right anterior | 45.16 | 4.20 | 46.85 | 5.69 | 44.47 | 5.88 | 44.86 | 5.73 |
| Right postlateral | 68.58 | 6.93 | 82.96 | 9.27 | 67.83 | 7.49 | 68.22 | 7.36 |
| Right postmedial | 69.14 | 7.86 | 82.42 | 8.49 | 66.59 | 6.55 | 66.99 | 6.40 |
| Right composite | 182.88 | 16.95 | 212.23 | 22.55 | 180.07 | 16.88 | 178.89 | 17.39 |
| Left anterior | 45.16 | 4.66 | 47.32 | 6.39 | 47.32 | 6.39 | 45.15 | 5.17 |
| Left postlateral | 66.96 | 8.34 | 82.81 | 9.43 | 67.01 | 6.81 | 67.01 | 6.81 |
| Left postmedial | 68.89 | 8.49 | 83.42 | 9.84 | 83.42 | 9.84 | 67.25 | 6.43 |
| Left composite | 181.02 | 19.83 | 213.55 | 24.63 | 179.79 | 14.91 | 178.62 | 15.29 |

**Data for Balance Error Scoring System**

|  | **NMT (n=22)** | | | | **CG (n=20)** | | | |
| --- | --- | --- | --- | --- | --- | --- | --- | --- |
|  | **Pretest** | | **Posttest** | | **Pretest** | | **Posttest** | |
|  | **Mean** | **SD** | **Mean** | **SD** | **Mean** | **SD** | **Mean** | **SD** |
| **Single Leg - Floor** | 3.55 | 0.91 | 6.6 | 2.46 | 2.5 | 1.06 | 5.85 | 2.21 |
| **Double Leg - Floor** | 3.86 | 1.36 | 5.35 | 1.84 | 2.5 | 0.96 | 4.25 | 1.86 |
| **Tandem - Floor** | 3.18 | 0.39 | 3.5 | 0.69 | 2.05 | 0.21 | 3.1 | 1.41 |
| **Single Leg - Foam** | 5.73 | 1.35 | 6.65 | 1.46 | 4.05 | 1.36 | 6.2 | 1.4 |
| **Double Leg - Foam** | 3 | 0.53 | 2.95 | 0.22 | 1.77 | 0.75 | 2.8 | 1.15 |
| **Tandem - Foam** | 3.14 | 0.35 | 2.95 | 0.22 | 2.41 | 0.73 | 2.95 | 1.19 |
